# Ultimate sensitivity in X-ray diffraction: angular moments versus shot noise

**DOI:** 10.1107/S1600576725006715

**Published:** 2025-08-28

**Authors:** Peter Modregger, Felix Wittwer, Ahmar Khaliq, Niklas Pyrlik, James A. D. Ball, Jan Garrevoet, Gerald Falkenberg, Alexander Liehr, Michael Stuckelberger

**Affiliations:** a Physics Department, University of Siegen, Germany; b Centre for X-ray and Nano Science CXNS Deutsches Elektronen-Synchrotron DESY Germany; c Physics Department, University of Hamburg, Germany; d ESRF, The European Synchrotron, Grenoble, France; e Deutsches Elektronen-Synchrotron DESY Germany; f Institute of Mechanical Engineering, University of Kassel, Germany; Diamond Light Source, United Kingdom

**Keywords:** X-ray diffraction, Bragg peak analysis, photon shot noise, photon counting detectors, angular moments

## Abstract

The sensitivity towards Bragg peak parameters constitutes a crucial performance attribute of X-ray diffraction experiments. Diffraction peaks are frequently characterized by model-free angular moment analysis, which offers greater versatility compared with traditional model-based peak fitting. Here, we have theoretically determined the ultimate limit of the sensitivities of angular moments imposed by photon shot noise. These predictions have been validated by experimental data provided by three different setups. We have observed experimentally achieved sensitivities of the first moment below 1/1000th of a detector pixel and below 1 µrad. The formulae provided for the uncertainties of angular moments can now be used to rapidly determine experimentally achieved sensitivities from a single diffraction frame. Corresponding limitations (*i.e.* ultra-low photon counts and exceedingly high photon count rates) are identified and discussed.

## Abstract 2

The ultimate shot-noise-limited sensitivity of model-free angular moment analysis for Bragg peak characterization in X-ray diffraction is theoretically determined and validated across three experimental setups. Uncertainty formulae to rapidly infer achievable sensitivities from a single diffraction frame are provided and limitations at ultra-low and high photon count rates are discusssed.

## 1. Introduction

Indisputably, Bragg peak analysis plays an essential role in X-ray diffraction (XRD) experiments. The different parameters of diffraction peaks provide insight to a large variety of sample properties, including material composition and identity of phases (via peak intensities), lattice parameters or internal strain and stress (via angular peak positions), and local crystal quality (via angular peak width) (Sedigh Rahimabadi *et al.*, 2020 ▸; Willmott, 2019 ▸). In standard data analysis, these parameters are extracted from 1D diffraction data by fitting to an appropriate model function. Some examples are Gaussian, Lorentzian or (pseudo)-Voigt functions (Harrington & Santiso, 2021 ▸).

However, if the model function employed deviates noticeably from the shape of the diffraction signal (*e.g.* double peaks or asymmetry), the fitted results can be ambiguous or misleading. Model-free peak analyses based on angular moments (also called centroid or center-of-mass methods) do not face this challenge and are, thus, more and more commonly used (Liu *et al.*, 2022 ▸; Ferrer *et al.*, 2023 ▸; Sjö *et al.*, 2025 ▸). For at least one possible application, *i.e.* the possibility for strain tensor tomography with XRD, peak data analysis based on angular moments is mandatory (Lionheart & Withers, 2015 ▸). Moment analysis is also used for other methods; examples include scanning small-angle X-ray scattering (Bunk *et al.*, 2009 ▸) and sub-pixel X-ray scattering (Modregger *et al.*, 2017 ▸).

An additional development in recent years is the rise of single photon counting detectors at synchrotron radiation beamlines dedicated to XRD (Leu *et al.*, 2016 ▸; Seo *et al.*, 2019 ▸; Wright *et al.*, 2020 ▸; Lu *et al.*, 2021 ▸; Chakrabarti *et al.*, 2022 ▸; Blankenburg *et al.*, 2023 ▸; Stone *et al.*, 2023 ▸). This type of detector offers a unique combination of high sensitivity, high dynamic range, and close to absent readout and dark current noise; some detector models also offer limited photon energy resolution (Trueb *et al.*, 2015 ▸). By counting single photons, these detectors cannot avoid photon shot noise, which obeys the well known Poisson statistics (Willmott, 2019 ▸).

In the following, we will demonstrate that the known statistics of single photon detectors allow for a theoretical prediction of the sensitivities of the first three angular moments of 1D diffraction peaks. We will validate the theory by comparison with experimental results from three different setups.

## 2. Theory

In this section we will derive the uncertainties (*u*) due to photon shot noise of the first three moments *M*_0_, *M*_1_ and *M*_2_ of a measured intensity distribution (*f*) using error propagation.

Suppose that a 1D intensity distribution, *f*, is measured as photon counts at equidistant sampling points, *x_j_*, with a separation of Δ*x*. The zeroth moment is given as (James, 2006 ▸)
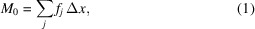
with *j* the number of the sampling position; the normalized first moment is 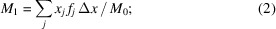
and, finally, the normalized and centralized higher moments of order *n* are 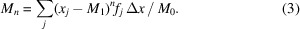
These moments are well defined for any experimentally obtained function, *f*. For sufficiently large sampling intervals and small sampling distances and in the absence of a background signal, the moments represent information about the underlying diffraction curve. Specifically, the zeroth moment corresponds to the integrated photon count, *N*_photons_, *i.e.*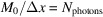. The first moment constitutes the center-of-mass (or centroid) position, which is identical to the peak position only for symmetric functions with a single peak. Otherwise, in some situations it can be useful to regard the first moment as the weighted sum of many contributing symmetric single peaks (Hauk, 1997 ▸). The square root of the second moment corresponds to the width of a peak shape function. For the frequently occurring case of a Gaussian function, the second moment is identical to the square of its standard deviation, *i.e.*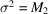.

The uncertainties of the photon count measurements, *f_j_*, due to photon shot noise are given by (Willmott, 2019 ▸) 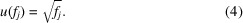
Each individual intensity measurement, *f_j_*, is affected by shot noise and the overall impact on the moments can be calculated using error propagation (Barlow, 1989 ▸): 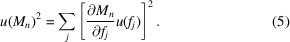
Applying this to the definition of moments [*i.e.* equations (1)–(3)] while considering the appropriate uncertainties, *i.e.* equation (4), it is straightforward to show that the uncertainties of the first three moments subjected to photon shot noise are given as 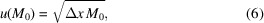

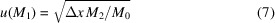
and 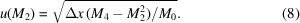
Details of the derivation are provided in Appendix *A[App appa]*. The results follow intuition: The zeroth moment constitutes the integrated photon counts and its absolute uncertainty increases while its relative uncertainty [*i.e.u*(*M*_0_/*M*_0_)] decreases with the number of collected photons. The uncertainty of the first moment decreases with the number of photons and increases with the width of the peak. The uncertainty of the second moment again decreases with the photon number while it increases with the difference between the fourth and the square of the second moment. Note that this difference is always positive as 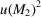 can be rewritten as 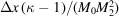 with the kurtosis κ, which obeys 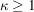 (James, 2006 ▸). Given sufficient photon counts, the above formulae can be used to connect moments observed in a single frame (*i.e.M*_0_, *M*_1_, *M*_2_ and *M*_4_) to information about the corresponding statistical ensembles [*i.e.u*(*M*_0_), *u*(*M*_1_) and *u*(*M*_0_)].

Evidently, experimentally encountered uncertainties may be larger than these results due to X-ray beam intensity fluctuations (*e.g.* intensity decay or injection), setup instabilities (*e.g.* drift of components) or non-linear response from the photon counting detectors. Some of these influences have been studied in the following experimental section. However, photon shot noise constitutes a fundamental limit, which renders the retrieved uncertainties the ultimate achievable sensitivities.

## 3. Results

The basic idea to determine experimental uncertainties follows a previous publication (Modregger *et al.*, 2011 ▸). Measurements are repeated a number of times and the standard deviations of resulting values are taken as uncertainties. The details of the utilized experimental setups play a minor role for the following analysis of the moment’s uncertainties. As long as the noise of detected signals follows shot noise statistics then the derived formulae for the uncertainties of angular moments are valid. As can be readily seen in the corresponding equations (6)–(8), the only contributing experimental parameters are the angular width of detector pixels (via Δ*x*), the total number of contributing photons (via *M*_0_), the width of the diffraction curves (via *M*_2_) and their kurtosis (via *M*_4_).

**Figure 1:**
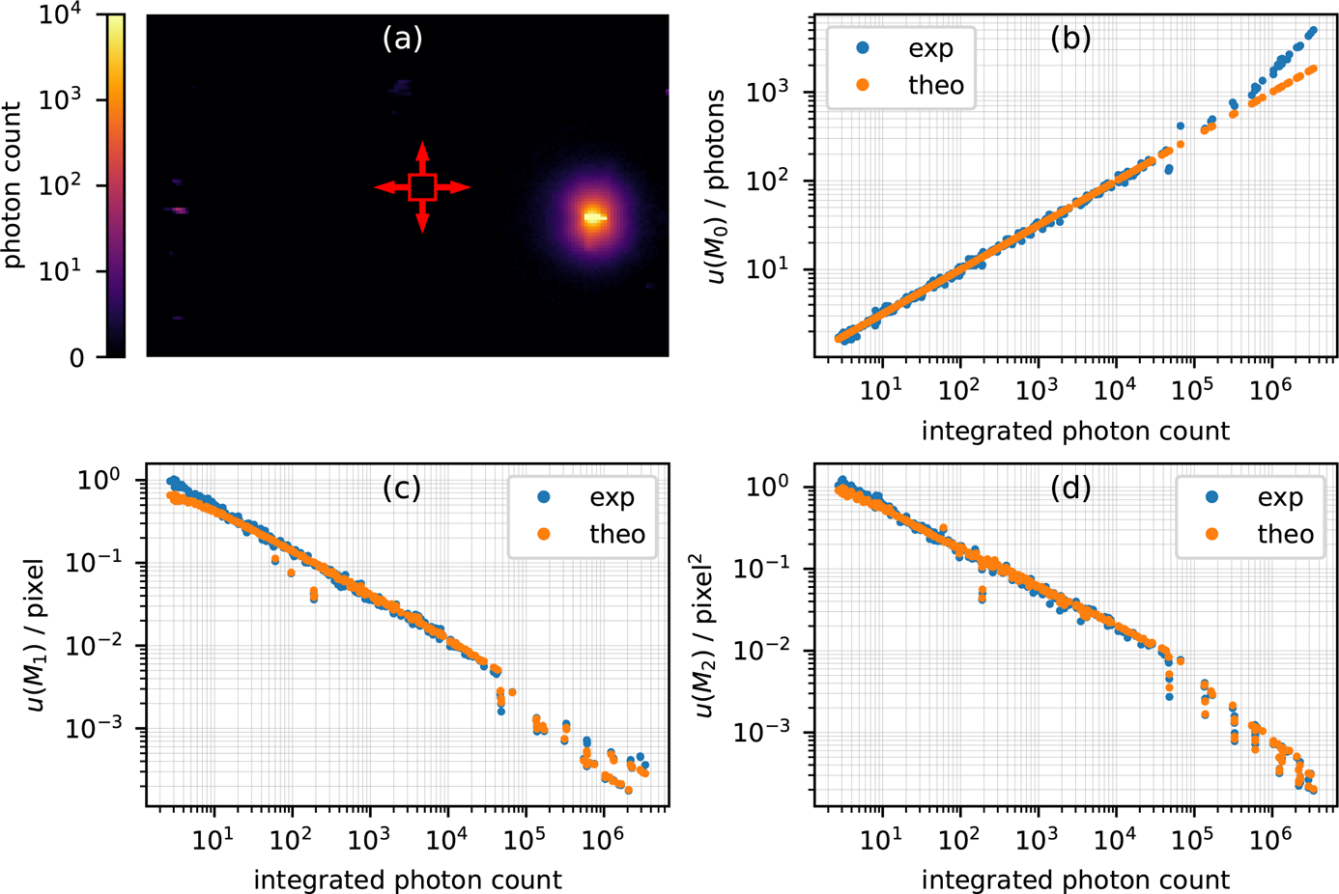
Moment uncertainties measured at P06, PETRA III. (*a*) Frame of a single diffraction spot. The rectangle and arrows indicate the window and its variation used for the following analysis. Uncertainty of (*b*) *M*_0_, (*c*) *M*_1_ and (*d*) *M*_2_ as a function of the total number of photons.

The first experiment was carried out at the P06 beamline of PETRA III in Hamburg, Germany (Schroer *et al.*, 2010 ▸; Falkenberg *et al.*, 2020 ▸). The setup was implemented for XRD with high spatial resolution similar to that described in the literature (Chakrabarti *et al.*, 2022 ▸; Khaliq *et al.*, 2024 ▸). A photon energy of 16.2 keV was selected by a double crystal monochromator. The beam was focused by Kirckpatrick–Baez mirrors to 510 nm in the horizontal and to 330 nm in the vertical direction, as measured by the X-ray fluorescence signal of a Pt stripe scanned through the beam. The sample, a 4H-SiC diode, was positioned on a 6-axis goniometer and the diffraction signal of the 0 0 0 12 reflection [see Fig. 1 ▸(*a*)] was collected by a Pilatus3 1M photon counting detector (DECTRIS, Switzerland) with a pixel size of 172 µm.

For noise analysis the measurement was repeated 100 times with an exposure time of 0.1 s. We determined the moments in terms of pixels implying Δ*x* = 1 pixel for equations (1)–(3) and (6)–(8). In order to determine the uncertainties of the moments as a function of both *M*_0_ (*i.e.* the total number of photons) and *M*_2_ (*i.e.* the width of the signal), we cut the signal to a sliding window with a size of 5 by 5 pixels, which is indicated by the red box in Fig. 1 ▸(*a*). The signal in the window was vertically summed resulting in an experimentally observed 1D function. As mentioned above, the moments are well defined for any experimentally obtained function including the described procedure for obtaining 1D data.

The total photon counts analyzed ranged over six orders of magnitude from 2.7 to 3.4 × 10^6^. This corresponded to photon count rates averaged over a data window of 1.1 cps (counts per second and pixel) to 1.4 × 10^6^ cps. The widths of the signals (*i.e.*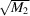) varied from 0.11 to 2.0 pixels. The experimental uncertainties were determined by the standard deviation of the moments of the individual 100 frames [*i.e.* standard deviation of equations (1)–(3)]. The theoretical uncertainties were determined by the average of the experimental moments and equations (6)–(8).

Fig. 1 ▸(*b*) compares the experimentally with the theoretically determined uncertainties for *M*_0_ showing a correlation coefficient of *r* = 0.98. The deviation for high photon counts was due to dead time effects for high count rates (Trueb *et al.*, 2015 ▸). Without any additional correction (as done here), the utilized detector shows a non-linear response above count rates of approximately 1 Mcps (Loeliger *et al.*, 2012 ▸). In the data at hand the highest observed count rate was 1.36 Mcps, which implies that the corresponding statistics did not follow pure photon statistics.

Fig. 1 ▸(*c*) shows the experimental and theoretical uncertainties for *M*_1_ with *r* = 0.95. While small deviations are visible at very high count rates, note that the first moment can be determined with an uncertainty below 1/1000th of a pixel. Larger deviations between theory and experiment have been observed for total photon counts below approximately 25. The known under-performance of the detector for rare 2 photon events may contribute to this deviation (Möller *et al.*, 2019 ▸). However, in some frames only a single photon was detected, and the corresponding zeroth moment is *M*_0_ = 1, *M*_1_ will be the pixel position of the counted photon and *M*_2_ will be 0. It seems obvious that from such a single frame no inference about the statistical ensemble can be drawn and equations (6)–(8) are not applicable anymore. Thus, we suggest to not use moment analysis in the case of very low photon numbers.

Fig. 1 ▸(*d*) demonstrates an equally high correlation of *r* = 0.97 between the experimentally determined and theoretically predicted uncertainties of the second moment. The small vertical sections are due to variations in the fourth moment as the data window varies over the diffraction peak.

**Figure 2:**
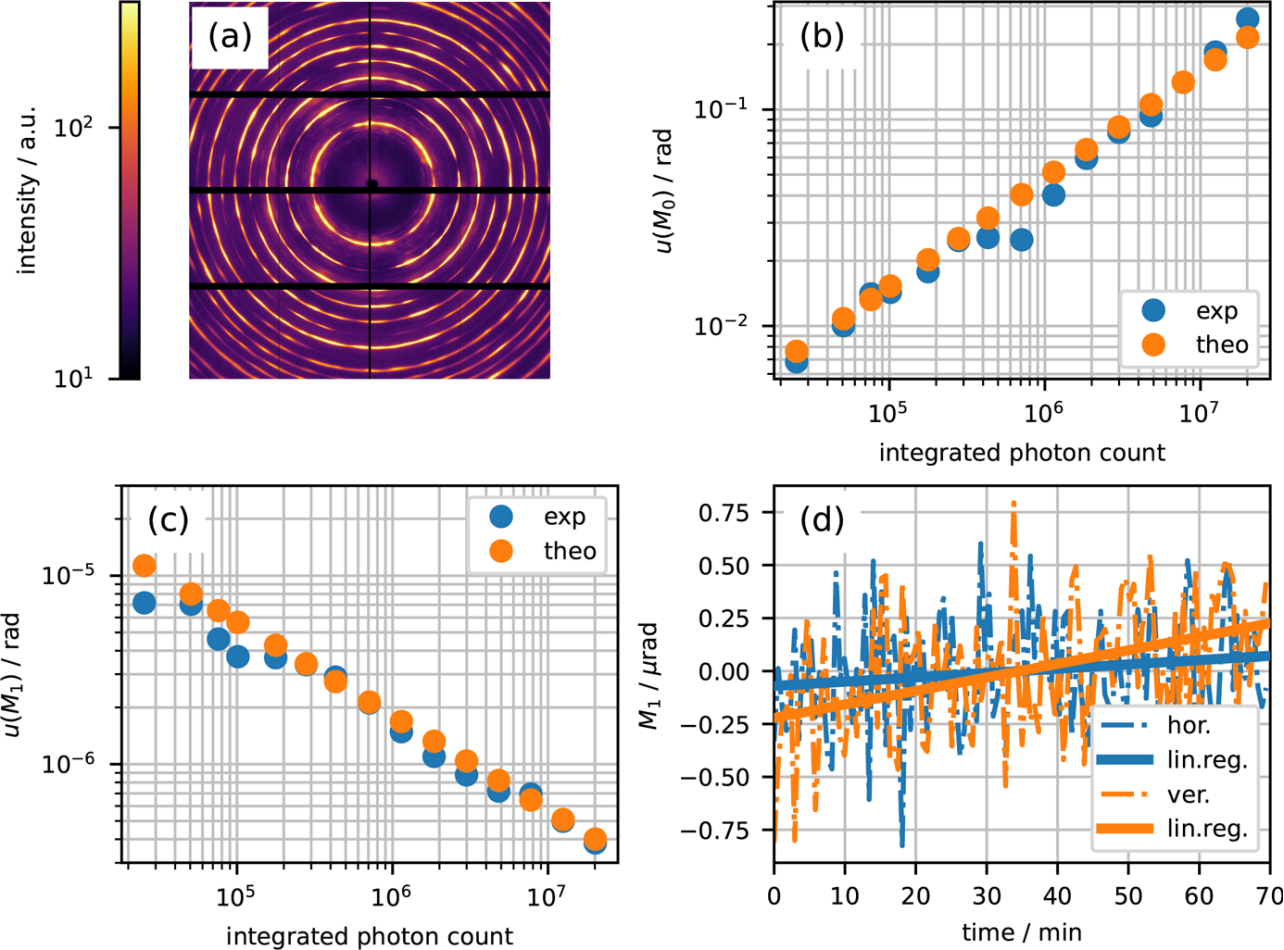
Sensitivity of XRD experiments at ID11 (ESRF). (*a*) Diffraction pattern of a martensitic steel sample acquired at the nanofocus station. Experimental and theoretical uncertainties of (*b*) the zeroth moment and (*c*) the first moment as a function of measured photon counts of the 321 diffraction ring with a Bragg angle of θ = 6.685°. (*d*) Horizontal and vertical angular setup stability measured over 70 min.

The second experiment was carried out at the nanofocus station of the ID11 beamline at the ESRF in Grenoble, France (Wright *et al.*, 2020 ▸). The horizontally aligned Si(111) double Laue monochromator provided a photon energy of about 70 keV. The X-ray beam was shaped by 32 Al compound refractive lenses for collimation and adjusted in size by perpendicular slits to 10 µm in the horizontal and 100 µm in the vertical direction. The sample was a martensitic steel rod with a diameter of 1 mm. The diffraction signal [see Fig. 2 ▸(*a*)] was collected by a photon counting Eiger2 X CdTe 4M detector (Dectris, Switzerland) about 0.3 m downstream of the sample. Calibration of the setup geometry was performed with a CeO_2_ calibrant and *pyFAI* (Kieffer *et al.*, 2020 ▸). The dataset will be available online (Khaliq *et al.*, 2027 ▸).

Two scans were performed. First, the diffraction pattern was measured with an exposure time of 2 ms 15800 times. The diffraction data were normalized and re-binned over repeated measurements to realize 20 independent instances of different effective exposure times ranging from 2 ms to 1.6 s. Each instance of the resulting diffraction patterns was then azimuthally integrated over the entire 321 ring using *pyFAI* to provide 1D diffraction curves. In order to retrieve the diffraction curves in terms of photon counts (and not ‘arbitrary’ intensities) after azimuthal integration, the diffraction patterns were normalized by 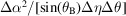, with Δα the angular pixel size, θ_B_ the Bragg angle, Δη the azimuthal range (here: 360°) and Δθ the angular resolution of the integration. This ensures that the zeroth moment corresponds to the integrated photon count, *i.e.*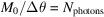. Experimental uncertainties for the zeroth and the first moment were then calculated from the standard deviation over instances of resulting moments. Figs. 2 ▸(*b*) and 2 ▸(*c*) demonstrate an excellent agreement between the experimental and theoretical uncertainties of *M*_0_ and *M*_1_ with correlation coefficients of *r* = 0.99 and *r* = 0.97, respectively.

Further, the angular stability of the setup was investigated with a second scan. This involved the repeated measurement of a diffraction pattern with 5 s exposure and a 30 s break, which covered a time span of about 70 min. The resulting first moments of the diffraction patterns integrated over 45° in the horizontal and vertical directions are shown in Fig. 2 ▸(*d*). Linear regression was used to provide upper bounds for the angular drift. In the horizontal direction, we determined a slope of 0.12 µrad h^−1^ with an *R*^2^ value of 0.16 and concluded that within the measurement error the horizontal direction is virtually drift free. In the vertical direction, we observed a slope of 0.38 µrad h^−1^ with an *R*^2^ value of 0.43, which indicates the presence of a small drift. Potential explanations for the vertical drift include a corresponding drift of the optical components upstream of the sample altering the X-ray beam trajectory or thermal/dose effects at the utilized detector. A sensitivity of the first angular moment below 1 µrad has been experimentally demonstrated.

**Figure 3:**
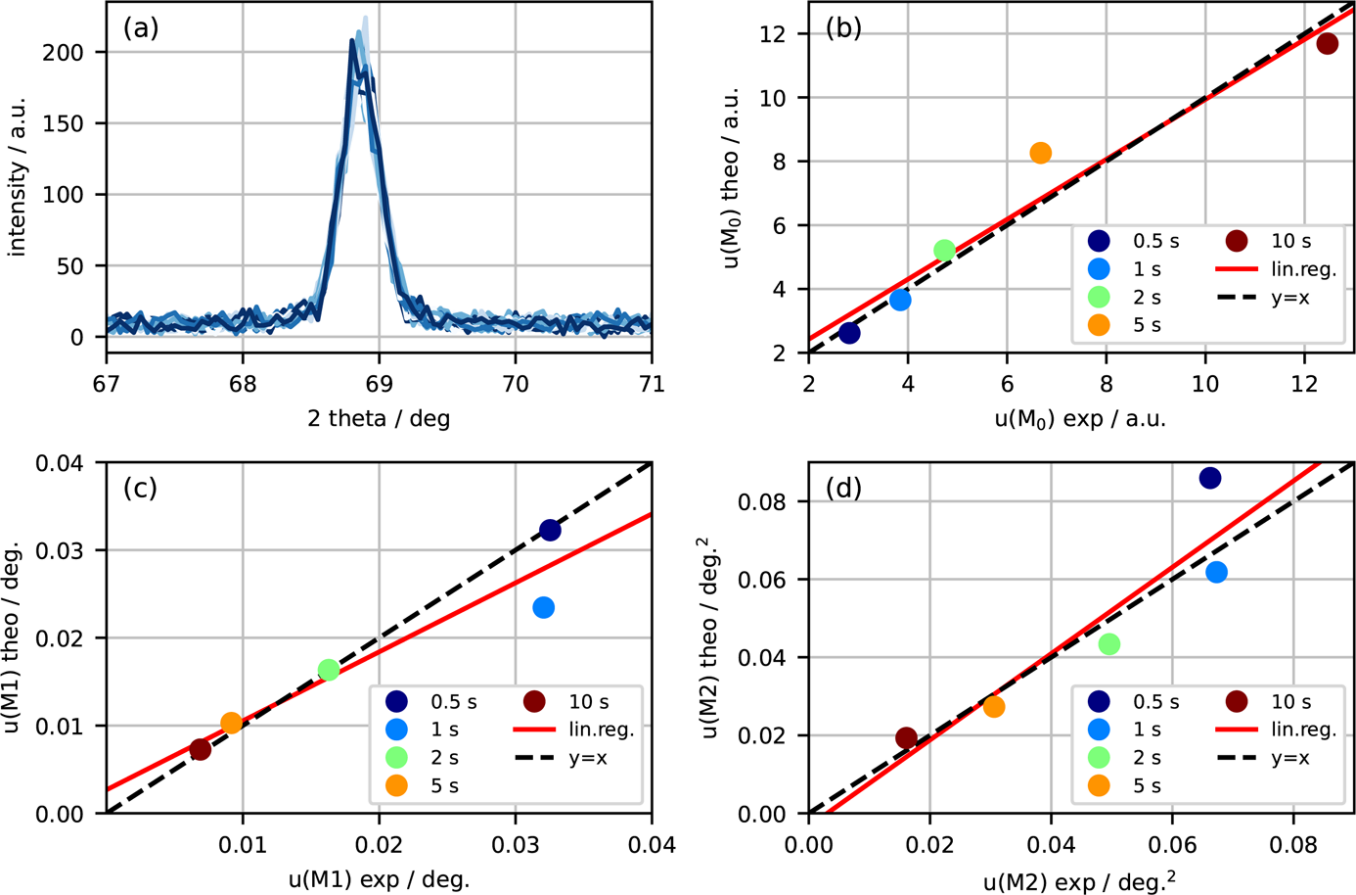
Uncertainties of moments measured with a laboratory XRD setup. (*a*) 30 diffraction curves of the 110 reflection of an α-Fe powder sample measured with an exposure time of 0.5 s. Scatter plots of resulting experimental and theoretical uncertainties for the (*b*) zeroth moment, (*c*) first moment and (*d*) second moment for several exposure times. Linear regression lines are shown in red, while the *y* = *x* line is shown as dashed black.

The third experiment was carried out using a laboratory XRD setup at the University of Kassel (Germany). The four circle diffractometer, manufactured by HUBER, was equipped with a chromium X-ray tube operated at an acceleration voltage of 35 kV and an electron current of 30 mA. Furthermore, a pinhole collimator with a diameter of 1 mm and a length of 112 mm was used to define the primary beam path. In the secondary beam path a slit system with a maximum divergence slit of 0.5°, a *K*β filter and a standard scintillator detector were installed. The 110 diffraction peak of a standard Fe powder was collected stepwise. The scan covered an angular interval of 2θ = ±2° with exposure times of 0.5, 1, 2, 5 and 10 s to realize the varying number of collected photons. For each exposure time the scan was repeated 30 times to measure the uncertainties [see Fig. 3 ▸(*a*)]. Since the utilized detector does not necessarily obey photon statistics, the Fano factor, χ (Fano, 1947 ▸), was determined for each 2θ data point by calculating 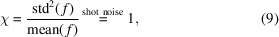
which, according to equation (4), is equal to 1 for a signal *f* that is affected only by photon shot noise. Here, the average ratio over scan points was χ = 1.06, which was considered sufficient for assuming dominating photon shot noise. We found a very good to excellent agreement between theoretically predicted and experimentally determined uncertainties for the zeroth moment [Fig. 3 ▸(*b*)] with a correlation coefficient of *r* = 0.97, for the first moment [Fig. 3 ▸(*c*)] with a correlation coefficient of *r* = 0.95 and for the second moment [Fig. 3 ▸(*d*)] with a correlation coefficient of *r* = 0.92.

## 4. Discussion

To demonstrate the universality of the derived formulae for the ultimate sensitivity limit of angular moments, we employed three distinct experimental setups. These differed in terms of the utilized X-ray source, type of detector, photon energies, photon spectra, photon flux and data analysis.

The P06 experiment used a Pilatus3 area detector to measure a single diffraction spot at 16.2 keV from a crystalline sample. Raw experimental frames were analyzed for the determination of achieved sensitivities. We found an upper limit in terms of photon counting rates (approximately 1 Mcps) for the applicability of the proposed framework, which resulted in significant deviations between theoretically predicted and experimentally determined sensitivities for *M*_0_. Further, we identified a lower limit of applicability at average photon counts below one. Both limits affected the sensitivities of *M*_1_ and *M*_2_, and we suggest using alternative data analysis approaches in this regime. Using the entire range of observed photon counts, the correlation coefficients between theory and experiment were still excellent with *r* = 0.98 for *u*(*M*_0_), *r* = 0.95 for *u*(*M*_1_) and *r* = 0.97 for *u*(*M*_2_). Sensitivities of the first moment below 0.1% of the pixel size and below 1 µrad have been observed, which underline the sub-pixel precision of this type of data analysis.

The ID11 experiment utilized an Eiger2 area detector, a powder diffraction signal at 70 keV with high total photon counts above 10^7^ and azimuthal integration of the acquired diffraction patterns as input for the sensitivity analysis. Observed correlation coefficients of *r* = 0.99 for *u*(*M*_0_) and *r* = 0.97 for *u*(*M*_1_) further validated our framework.

Finally, the laboratory-based experiment used a scintillator point detector and an X-ray tube providing a broader spectrum. In addition, the acquisition of repeated measurements involved the movement of motors, which was not the case for the two previous experiments. The applicability of photon shot noise was confirmed by determination of the average Fano factor (χ = 1.06). The correlation coefficients were slightly smaller compared with the synchrotron-based experiments, with *r* = 0.97 for *u*(*M*_0_), *r* = 0.95 for *u*(*M*_1_) and *r* = 0.92 for *u*(*M*_2_). We attribute this to the limited (re)positioning accuracy of the scanning motors, which constitute an additional source of noise.

## 5. Conclusions

The photon shot noise limited uncertainties of angular moments have been theoretically derived and compared with experimentally determined values. Theoretical predictions have been validated by very high correlation coefficients for different experimental setups and data analysis procedures. The formulae for the uncertainties [*i.e.* equations (6)–(8)] can now be used to rapidly determine expected sensitivities from single diffraction frames. For example, a frame taken from the ID11 dataset with an exposure time of 20 ms and with the 321 reflection integrated over an azimuthal range of (−45°, +45°) resulted in a diffraction curve with approximately 65000 photons and a second moment of 2 × 10^−6^ rad^2^. The corresponding sensitivity for the first moment according to equation (7) is approximately 6 µrad, which is in agreement with the result shown in Fig. 2 ▸(*c*). The formulae provided could also be used to significantly speed up numerical simulation aimed at the determination of the influence of photon shot noise.

## Supplementary Material

X-ray powder diffraction data of martensitic steel acquired at the ID11 beamline of the ESRF.: https://doi.org/10.15151/ESRF-ES-1837447580

## Appendix A Derivation of uncertainties

In the following the details of the derivation of the uncertainties of the first three moments subjected to photon shot noise are given. For this it is convenient to first calculate the following partial derivatives, starting with the zeroth moment defined in equation (1):

where , Kronecker’s δ was used. The corresponding partial derivative for the first moment, given in equation (2), is

where equation (10) was used and which can be simplified to

The derivative of the second moment, *i.e.* equation (3) with *n* = 2, requires a bit more work:

Applying the derivative leads to

where again equation (10) was used. Identifying the occurring term *M*_0_*M*_2_ in the last summand and using equation (12) yields

The remaining term with the derivative is

where it is straightforward to show that the occurring sum,

cancels to zero. Thus, the partial derivative of the second moment is given by

Now, using the uncertainty in photon counting measurements, *i.e.* equation (4), and the formula for error propagation, *i.e.* equation (5), the uncertainties of the first three moments are straightforward to calculate. Beginning with the zeroth moment and using equation (10) retrieves the desired result:

For the first moment utilizing equation (12) we have

Finally, the uncertainty of the second moment is

which can be rearranged to

where the fourth moment, *i.e.* equation (3) with *n* = 4, occurs. This can be further simplified to

Quod erat demonstrandum.
